# Insight into the Critical Role of Exchange Current Density on Electrodeposition Behavior of Lithium Metal

**DOI:** 10.1002/advs.202003301

**Published:** 2021-01-06

**Authors:** Yangyang Liu, Xieyu Xu, Matthew Sadd, Olesya O. Kapitanova, Victor A. Krivchenko, Jun Ban, Jialin Wang, Xingxing Jiao, Zhongxiao Song, Jiangxuan Song, Shizhao Xiong, Aleksandar Matic

**Affiliations:** ^1^ State Key Laboratory for Mechanical Behavior of Materials Xi'an Jiaotong University Xi'an 710049 P. R. China; ^2^ Faculty of Materials Science Lomonosov Moscow State University Leninskie Gory 1 Moscow 119991 Russia; ^3^ Department of Physics Chalmers University of Technology Göteborg SE‐412 96 Sweden; ^4^ Institute of Arctic Technology Moscow Institute of Physics and Technology 9 Institutskiy per., Dolgoprudny Moscow 141701 Russia

**Keywords:** electrochemical kinetics, electrodeposition, exchange current density, Li metal, phase‐field model

## Abstract

Due to an ultrahigh theoretical specific capacity of 3860 mAh g^−1^, lithium (Li) is regarded as the ultimate anode for high‐energy‐density batteries. However, the practical application of Li metal anode is hindered by safety concerns and low Coulombic efficiency both of which are resulted fromunavoidable dendrite growth during electrodeposition. This study focuses on a critical parameter for electrodeposition, the exchange current density, which has attracted only little attention in research on Li metal batteries. A phase‐field model is presented to show the effect of exchange current density on electrodeposition behavior of Li. The results show that a uniform distribution of cathodic current density, hence uniform electrodeposition, on electrode is obtained with lower exchange current density. Furthermore, it is demonstrated that lower exchange current density contributes to form a larger critical radius of nucleation in the initial electrocrystallization that results in a dense deposition of Li, which is a foundation for improved Coulombic efficiency and dendrite‐free morphology. The findings not only pave the way to practical rechargeable Li metal batteries but can also be translated to the design of stable metal anodes, e.g., for sodium (Na), magnesium (Mg), and zinc (Zn) batteries.

## Graphical abstract

The effect of exchange current density on electrodeposition behavior of lithium (Li) is revealed by a phase‐field modeling and validated by experimental data. The results show that lower exchange current density contributes to uniform distribution of cathodic current density and formation of nuclei with larger critical radius, two factors that promote dense Li deposition, high Coulombic efficiency, and dendrite‐free morphology.

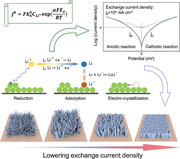

## 1 Introduction

With the highest theoretical capacity of 3680 mAh g^−1^, the lowest reduction potential (–3.04 V vs standard hydrogen electrode), and low density of 0.53 g cm^−3^, metallic lithium (Li) is considered as the ultimate anode material for pursuing high‐energy‐density batteries.^[^ ^1^ ^]^ When coupled with high‐capacity cathodes, such as sulfur or oxygen, the Li metal battery (LMB) systems can meet the demand of energy density beyond 500 Wh kg^−1^.^[^ ^2^, ^3^ ^]^ However, the commercialization of LMBs has been hindered by a low Coulombic efficiency and the safety concerns, which are connected to the electrochemical plating process with unavoidable growth of Li dendrites and a high reactivity of metallic Li with the common electrolytes used.^[^ ^2^ ^]^ The dendritic morphology of deposited Li enlarges the reactive area towards the electrolyte and thus generates a larger solid electrolyte interphase (SEI) by which both electrolyte and active Li will be consumed.^[^ ^4^ ^]^ In addition, the dendritic Li covered by an inert SEI can easily break into fragments by internal stresses generating “dead Li.”^[^ ^5^ ^]^ This electronically isolated “dead Li” cannot participate in electrochemical reactions and resulting in a low Coulombic efficiency.^[^ ^6^ ^]^ The safety concerns, on the other hand, result from Li dendrites penetrating through the separator leading to internal short‐circuit and potential thermal runaway of the battery as a consequence.^[^ ^1^ ^]^

To tackle the above issues, a variety of strategies to suppress the growth of Li dendrites and weaken the side‐reactions have been exploited,^[^ ^3^, ^7^, ^8^ ^]^ including building an artificial SEI on Li metal,^[^ ^9^ ^]^ designing Li composite structures,^[^ ^10^, ^11^, ^12^ ^]^ optimization of electrolyte,^[^ ^13^, ^14^ ^]^ and modification of separator or Li surface.^[^ ^15^, ^16^, ^17^ ^]^ In spite of these advancements, the electrochemical performance is still far from the practical requirements for utilization of Li metal anodes that is highly dependent on an ultrahigh Coulombic efficiency (>99.8%) over extended cycling and a dendrite‐free, dense, morphology after electrochemical plating.^[^ ^18^ ^]^ Thus, fundamental understanding of the mechanism of Li dendrite growth during the electrochemical plating‐stripping processes is crucial to guide design strategies for LMBs. So far, research works on the fundamental understanding of Li metal anode have been reported by using advanced characterization of Li surfaces,^[^ ^19^ ^]^ and theoretical simulations.^[^ ^20^, ^21^, ^22^, ^23^ ^]^ Several models have been developed to describe the electrodeposition behavior of Li, including surface nucleation models invoking surface energy,^[^ ^24^, ^25^ ^]^ space charge model,^[^ ^26^, ^27^ ^]^ and the mass‐transfer model.^[^ ^28^, ^29^, ^30^ ^]^ Unfortunately, the instructive clues that can guide the research to tackle the problematic issues of Li metal anodes are still insufficient.

On account of fundamental electrochemistry, the electrochemical deposition process of Li can be divided into two interdependent steps, the reduction of a Li ion (Li^+^) to form a single Li atom and the electrocrystallization process of Li atoms.^[^ ^31^, ^32^ ^]^ For a Li electrode at an equilibrium potential for the Li^+^/Li redox couple, a dynamic situation on the electrode surface is formed by the reduction of Li^+^ to Li and the oxidation of Li to Li^+^ that both occur with the same rate, just as for equilibrium states in other chemical processes. The current density at equilibrium can be written as *j* = *j*_a_ + *j*_c_ = 0, where *j*_a_ and *j*_c_ are the partial anodic and cathodic current densities, respectively. The magnitude of these partial current densities at equilibrium, which is an indicator of the electron‐transfer activity on the electrode surface at the equilibrium potential, has be identified as an intrinsic kinetic parameter and is known as the exchange current density,^[^ ^33^ ^]^ *j* ^0^ = *j*_a_ = −*j*_c_. A higher exchange current density means that extensive oxidation and reduction reactions occur while a low value indicates the opposite. In the case of Li deposition, the exchange current density is a crucial parameter to understand the intrinsic kinetics of the electron‐transfer activity on the electrode for the reduction process of Li^+^ to Li, but has not attracted enough attention in research on LMBs so far.

In this work, we have developed a model to describe the role of the exchange current density for the electrodeposition behavior of Li metal electrodes. The model is built on the Fick's law and Butler–Volmer equation by using a phase‐field method combined with a random walk algorithm.^[^ ^34^ ^]^ Therefore, this phase‐field model is capable of simulating the kinetic behavior of electrochemical processes, such as electrodeposition of Li, which is difficult to access by other modeling methods. The modeling work is complemented by comprehensive experimental data, such as properties of a series of electrolytes, performance, and morphology of Li electrodes, acquired as input parameters for modeling and/or to validate the model. With the combined perspective of modeling and experiments, we show that a low exchange current density on the Li electrode surface will result in a columnar structure of deposited Li with low aspect ratio that will promote a dense electrodeposition of Li with high Coulombic efficiency and dendrite‐free morphology.

## 2 Results and Discussion

### 2.1 Relation between Exchange Current Density and Electrodeposition

**Figure 1:**
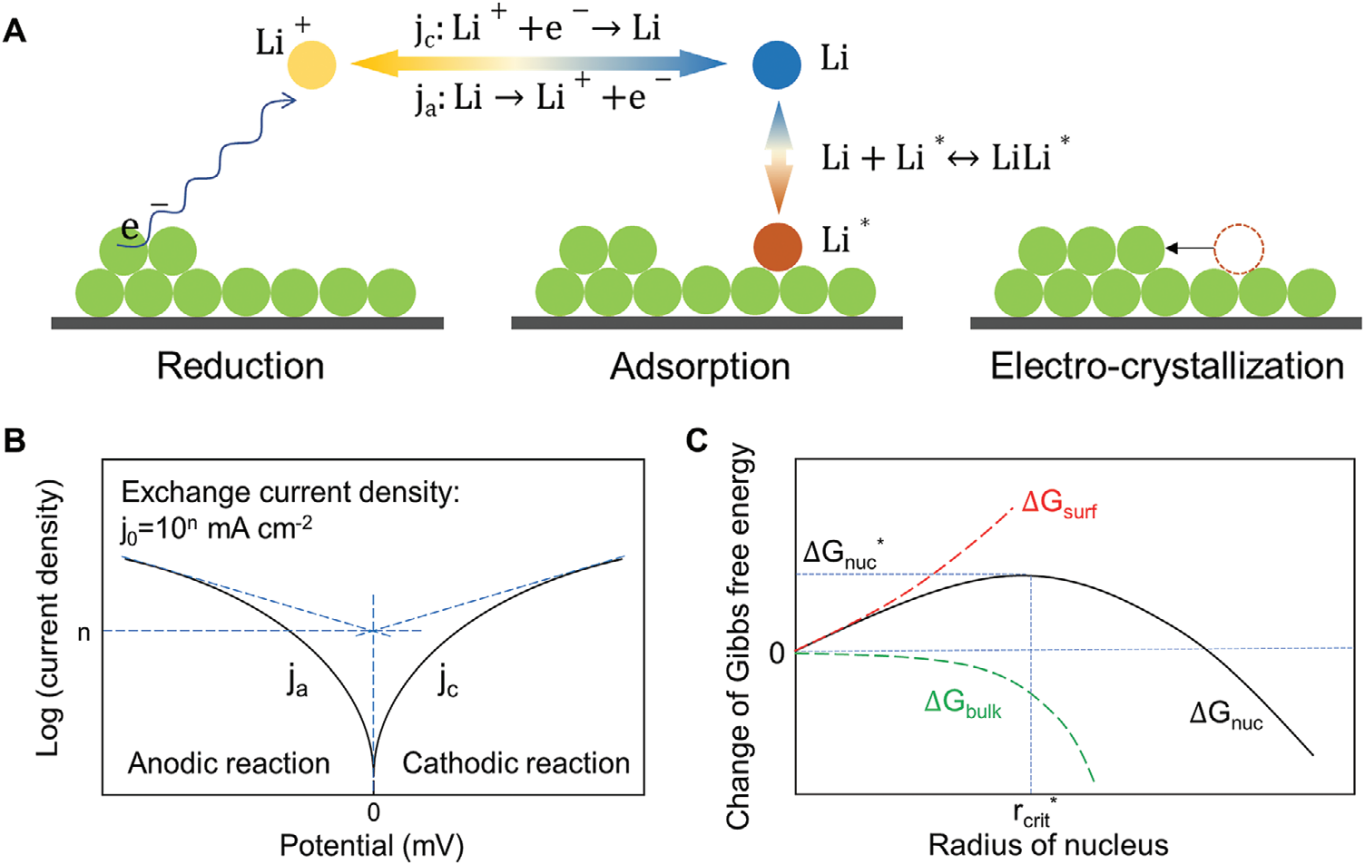
Schematic illustration of A) the electrodeposition process of Li metal, B) Tafel curve and the estimation of *n* to calculate the exchange current density, and C) Gibbs free energy change as a function of radius of a nucleus.

As discussed above, electrodeposition of Li can be divided into two steps after the mass transfer of Li ion through electrolyte and its dissociation from its solvation shell, reduction of Li ions to form adsorbed Li atoms and the following electrocrystallization, as show in **Figure** **1**A.^[^ ^31^, ^32^ ^]^ In the first step, the reduction of Li ion, Li^+^ + *e* ^−^⇌Li, results in a current density at a certain potential for the single‐electron reaction that is given by the Butler–Volmer equation^[^ ^35^, ^36^, ^37^ ^]^

(1) $$j\;=j^{0}\;\left[\frac{C_{Li^{+}}^{s}}{C_{Li^{+}}^{b}}\exp\left(\frac{\alpha F}{RT}\eta \right)-\frac{C_{\mathrm{Li}}^{s}}{C_{\mathrm{Li}}^{b}}\exp\left(-\frac{\beta F}{RT}\eta \right)\right]$$

where $C_{Li^{+}}^{b}$ and $C_{Li^{+}}^{s}$ are the concentrations of Li ion in bulk electrolyte and on the Li electrode surface, respectively. *α* and *β* are the anodic and cathodic transfer coefficient (*α* + *β* = 1 for single electron transfer), respectively. *F* is the Faraday's constant and *R* is the ideal gas constant. *T* is Kelvin temperature and *η* is overpotential of reduction. According to Equation (1), the overpotential is the unambiguous driving force of the electrochemical reaction.

Because of a high polarization of the electrode surface in LMBs, i.e., large *η*, the second term in Equation (1), $\exp(-\frac{\beta F}{RT}\eta )$, can be neglected resulting in^[^ ^38^ ^]^

(2) $$j\approx j^{0}\frac{C_{Li^{+}}^{s}}{C_{Li^{+}}^{b}}\exp\left(\frac{\alpha F}{RT}\eta \right)$$

This equation shows that at a certain overpotential, the cathodic current density of the reduction process is directly proportional to the exchange current density (*j* ^0^).^[^ ^39^ ^]^ To obtain the exchange current density, Equation (2) can be transformed to

(3) $$\eta =-\frac{2.3RT}{\alpha F}\mathrm{lg}\left(\frac{C_{Li^{+}}^{s}}{C_{Li^{+}}^{b}}j^{0}\right)+\frac{2.3RT}{\alpha F}\mathrm{lg}j$$

which is the Tafel equation. The Tafel equation can be drawn as the Tafel curve in which the exchange current density value can be calculated as shown in Figure 1B.^[^ ^40^ ^]^

Following the reduction process, the formed Li atom will be adsorbed to a kink position at the surface and the electrocrystallization of Li subsequently occurs.^[^ ^41^ ^]^ This process can be divided into an initial nucleation and the following growth, which are determined by the thermodynamics on the electrode surface and the mass transfer near the surface, respectively (Figure 1C). The Gibbs free energy change of nucleation (Δ*G*_nuc_) can be described as^[^ ^42^, ^43^ ^]^

(4) $$\Delta G_{\mathrm{nuc}}=-\frac{2\pi }{3}r^{3}\frac{\Delta G_{B}}{V_{m}}+2\pi r^{2}\sigma$$

where Δ*G*_B_ is the Gibbs free energy change when a Li atom is adsorbed to the surface compared to being in the supersaturated electrolyte, *V*_m_ is the molar volume of Li metal, *r* is the radius of semi‐sphere nuclei, while *σ* is the surface tension between the Li nuclei and the electrolyte. Thus, the Δ*G*_nuc_ is strongly related to the radius of the nucleus, and a maximum in Δ*G*_nuc_ appears when *r* reaches a critical value (critical radius of the nucleation, *r*_crit_). In an electrochemical system, Δ*G*_B_ is related to the overpotential (*η*) through

(5) $$\Delta G_{B}=-nF\left|\eta \right|$$

and Equation (4) can be written as^[^ ^43^, ^44^, ^45^ ^]^

(6) $$\Delta G_{\mathrm{nuc}}=-\frac{2\pi }{3}r^{3}\frac{-nF\left|\eta \right|}{V_{m}}+2\pi r^{2}\sigma$$

Therefore, the nucleation process is also associated to the exchange current density through the overpotential (Equations (3) and (6)). Thus, the exchange current density is a crucial parameter for the kinetics of the two steps of the electrodeposition process of Li.

### 2.2 Electrodeposition of Li in Electrolytes with Tunable Properties

**Figure 2:**
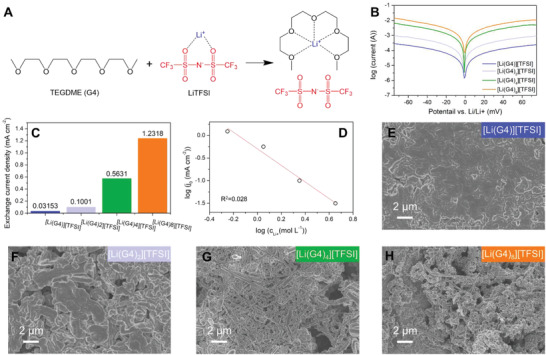
Experimental results for electrodeposition behaviors of Li in four electrolytes based on same solvent and Li salt. A) Chemical structures of TEGDME (G4), LiTFSI, and the formed [Li(G4)]^+^ [TFSI]^−^ solvate complex. B) Tafel curves for Li electrode in different electrolytes and C) corresponding exchange current densities derived from Tafel curves. D) Relationship between the concentration of Li ions and exchange current density. E–H) SEM images of Li anodes cycled in the electrolytes E) [Li(G4)][TFSI], F) [Li(G4)_2_][TFSI], G) [Li(G4)_4_][TFSI], and H) [Li(G4)_8_][TFSI]. All Li anodes were retrieved from Li||Li symmetric cells cycled at a current density of 0.5 mA cm^−2^ and an area capacity of 0.5 mAh cm^−2^ for 50 cycles.

To perform a reliable modeling of electrodeposition behavior of Li, parameters derived from a series of electrolytes and the electrochemical performance of Li electrode in them are acquired. The value of exchange current density derived for each electrolyte is used as input data for the modeling and the performance of Li electrode, such as Coulombic efficiency, cycling life, and morphology after cycling, is used to validate the results of modeling. The electrolyte is systematically designed using the same solvent G4 (tetra ethylene glycol dimethyl ether (TEGDME)) and the same Li salt, lithium bis(trifluoromethanesulfonyl)imide (LiTFSI), but with various stoichiometric ratios, in which LiTFSI/G4 forms a coordinating complex (**Figure** **2**A). The local structure of these electrolytes is highly dependent on the G4‐to‐Li^+^ ratio and we have previously reported that the number of contact ion pairs increases, and solvent‐separated ion pairs decrease, when the ratio of G4 to Li^+^ is lowered.^[^ ^46^ ^]^ The equimolar electrolyte is well known as a solvate ionic liquid (SIL) or highly concentrated electrolyte (HCE) and has been shown to enable relatively stable cycling of Li metal anodes.^[^ ^47^, ^48^, ^49^ ^]^

In this work, we tune the G4/Li^+^ ratio from 1 to 8, forming [Li(G4)*_n_*][TFSI] electrolytes (*n* = 1, 2, 4, 8) with exactly same chemical components but a broad variation of physical properties like conductivity, viscosity, and density, which means that only the physical properties of the electrolyte are used in our simulation and the results are not dependent on the actual chemistry. These parameters are strongly related to the mass transfer process at the electrode surface, resulting in a tunable exchange current density as a function of electrolyte. As shown in Figure 2C, the exchange current density derived from the Tafel curves in Figure 2B increases dramatically with *n* for the [Li(G4)*_n_*][TFSI] electrolytes. The influence of the SEI is not excluded by the analysis of the Tafel curves since the SEI is formed on Li metal as soon as it gets in contact with the electrolyte. A linear relation between the logarithm of exchange current density and the logarithm of molar Li‐ion concentration is revealed in Figure 2D. All physical parameters and electrochemical properties for each electrolyte are summarized in Table S1, Supporting Information.

To evaluate the impact of electrolytes properties on the electrodeposition behavior of Li electrodes, galvanostatic plating/stripping tests in both Li||Li symmetric cells and Li||Copper (Cu) half‐cells, electrochemical impedance spectroscopy (EIS), and scanning electron microscopy (SEM) experiments were carried out. The results in Figures S2 and S3, Supporting Information, show that Li electrodes cycled in the [Li(G4)_4_][TFSI] and [Li(G4)_8_][TFSI] electrolytes have unstable interfaces and rapidly increasing interfacial resistances compared to the cells using [Li(G4)_2_][TFSI] and [Li(G4)][TFSI] electrolytes. Furthermore, Coulombic efficiency of the plating/stripping process, which is a crucial parameter to estimate the reversibility of active Li, is up to 97% after 20 cycles with the [Li(G4)][TFSI] electrolyte, i.e., the SIL, but is considerably lower for the other electrolytes and decreases with decreasing Li‐ion concentration (Figure S4, Supporting Information). It is generally accepted that the low Coulombic efficiency is attributed to dendritic growth of Li upon plating and the collapse of these structures during Li stripping leads to the disconnection of active Li from the bulk electrode, or current collector, generating inactive (“dead”) Li that corresponds to the capacity loss.^[^ ^50^ ^]^ This scenario is also underlined by the SEM images shown in Figure 2E–H. The deposited Li in the [Li(G4)_8_][TFSI] and [Li(G4)_4_][TFSI] electrolytes shows needle‐like dendritic morphology while a denser structure is obtained when Li is deposited from the [Li(G4)_2_][TFSI] electrolyte. It is worth noting that the Li deposited from the [Li(G4)][TFSI] electrolyte (Figure 2E) shows an extremely flat and dendrite‐free morphology with few pores, indicating a superior microstructure for stable Li deposition and potentially high Coulombic efficiency.

The above results suggest that the electrodeposition behavior in these electrolytes is strongly affected by the physicochemical properties here tuned by the solvent‐to‐Li salt ratio, which is consistent with previous results in literatures.^[^ ^49^, ^50^ ^]^ However, the underlying mechanism for how these physicochemical parameters, in particular the exchange current density, affect the electrochemical processes on Li electrodes is still unclear. To provide the connection through a thermodynamic perspective, we have performed phase‐field modeling to investigate the relationship between the exchange current density and the time‐dependent electrochemical process on a Li electrode using the COMSOL Multiphysics 5.5 platform.

### 2.3 Distribution of Local Current Density on the Li Surface

**Figure 3:**
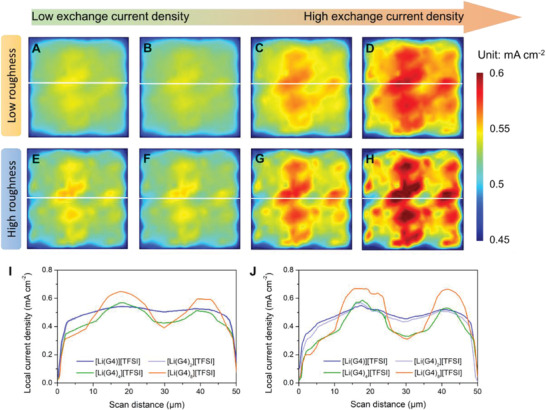
Distribution of local current density on a low‐roughness Li electrode (50 µm × 50 µm) when depositing Li from the electrolytes A) [Li(G4)][TFSI], B) [Li(G4)_2_][TFSI], C) [Li(G4)_4_][TFSI], and D) [Li(G4)_8_][TFSI], as well as that on the high‐roughness Li electrode (50 µm × 50 µm) from the electrolytes E) [Li(G4)][TFSI], F) [Li(G4)_2_][TFSI], G) [Li(G4)_4_][TFSI], and H) [Li(G4)_8_][TFSI]. Applied current density is 0.5 mA cm^−2^. I,J) Line profiles of local current density corresponding to the white lines in (A–D) and (E–H), respectively.

To reveal the distribution of local current density, which is corresponding to the rate of reduction of Li ions locally on the surface of Li electrode, we constructed large‐scale model substrates (50 µm × 50 µm) with low and high roughness, respectively, as shown in Figure S5, Supporting Information. An overall area current density of 0.5 mA cm^−2^ is applied until the equilibrium state is reached. The results in **Figure** **3**A–D show that the distribution of local current density is highly uneven on the low‐roughness surface of Li substrate and this nonuniformity is exacerbated by a higher ratio of G4 to LiTFSI, i.e., by increasing the exchange current density for Li depositing in the electrolyte. Line profiles, corresponding to the location of the line in each figure, are shown in Figure 3I. The highest local current densities along the profiles are 0.54, 0.55, 0.57, and 0.65 mA cm^−2^ on the Li substrate in [Li(G4)][TFSI], [Li(G4)_2_][TFSI], [Li(G4)_4_][TFSI], [Li(G4)_8_][TFSI], respectively. The corresponding local current densities on the high‐roughness Li substrate (Figure 3E–H) are 0.55, 0.56, 0.58, and 0.67 mA cm^−2^. A similar distribution is observed at higher applied current densities (1.0 mA cm^−2^, Figure S6, Supporting Information). The inhomogeneity of local current density on the Li substrate will result in preferred deposition on spots with higher local current density that not only induces dendrite growth, but will generate cracks in the protective SEI film and thus fresh metallic Li may react with the electrolyte leading to loss in electrochemical activity and overall capacity.^[^ ^7^, ^51^ ^]^ Therefore, a lower exchange current density results in a more even distribution of local current density on the surface of the electrode and more uniform electrodeposition of Li, with lower capacity loss as a consequence.

### 2.4 Nucleation of Li on the Electrode Surface

**Figure 4:**
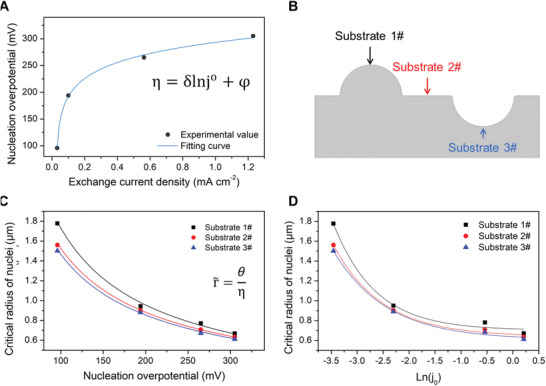
A) Nucleation overpotential of Li as a function of exchange current density. B) Schematic of the nucleation substrate with different topography. C) Critical radius of nucleation on different substrates as a function of nucleation overpotential. D) Critical radius of nucleation on different substrates as function of exchange current density.

In the electrodeposition process, the reduction of Li^+^ is followed by electrocrystallization, with the nucleation of Li and the subsequent growth and this determines the morphology of the deposited Li. To reveal the effect of exchange current density on the nucleation process, we measured the overpotential of Li nucleation on Cu current collectors for the series of electrolytes and the results, derived from the voltage profiles (Figure S7, Supporting Information), are shown as a function of exchange current density in **Figure** **4**A. In an experiment, the overpotential is a negative number and its absolute value is plotted here. The data fit well to the transformed form of Equation (2)

(7) $$\eta =\delta \ln j^{0}+\varphi \left(\delta =-\frac{RT}{\alpha F}\;\text{and}\;\varphi =\frac{RT}{\alpha F}\;\ln\frac{jC_{Li^{+}}^{b}}{C_{Li^{+}}^{S}}\right)$$

showing that the increase of exchange current density contributes to a higher overpotential for Li nucleation. To calculate the critical radius of nucleation of Li depositing in different electrolytes and for different morphologies, three substrates, including semi‐circle bulge (1#), perfect plate (2#), and semi‐circle depression (3#) (Figure 4B), were constructed. After solving equation

(8) $$\frac{d\Delta G_{\mathrm{nuc}}}{dr}=0$$

the dependence of the critical radius ($\tilde{r}$) on the overpotential is given by

(9) $$\tilde{r}=-\frac{\theta }{\left|\eta \right|},\;\text{where}\;\theta =\frac{2\sigma V_{m}}{nF}\;$$

and the results are summarized in Table S2, Supporting Information, and plotted in Figure 4C. Combining Equations (6) and (8), the critical radius ($\tilde{r}$) can be expressed as a function of exchange current density

(10) $$\tilde{r}=-\frac{\theta }{\left|\delta \ln j^{0}+\varphi \right|}$$

As shown in Figure 4D and Figure S8, Supporting Information, a lower exchange current density on the Li electrode leads to an increase of the critical radius for Li electrocrystallization. The obtained data for Li nucleation are further used to model the growth process.

### 2.5 Growth of Deposited Li

**Figure 5:**
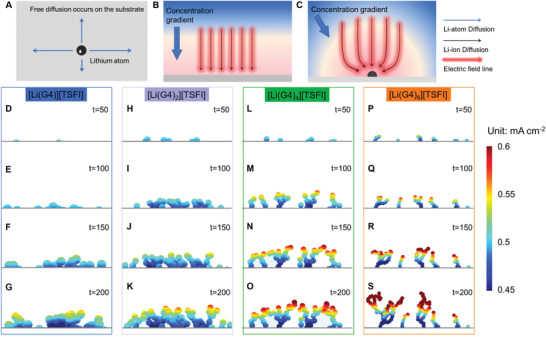
A) Free diffusion of Li on the surface of the substrate. B,C) Diffusion of Li ions in the bulk of electrolyte is controlled by the coupled concentration gradient and electric field near the substrate B) without and C) with structural heterogeneities. Simulated morphology with distribution of local current density for Li deposited in the electrolytes D–G) [Li(G4)][TFSI], H–K) [Li(G4)_2_][TFSI], L–O) [Li(G4)_4_][TFSI], and P–S) [Li(G4)_8_][TFSI]. Applied current density is 0.5 mA cm^−2^.

To investigate the electrochemical process of Li growth as a function of exchange current density, phase‐field modeling coupled with a random walk algorithm was performed on a substrate allowing free diffusion of Li, as shown in **Figure** **5**A. Meanwhile, the diffusion of Li ion in the bulk electrolyte near the substrate is controlled by the multi‐physical field including a concentration gradient and an electric field. A uniform concentration and electric field are obtained for a flat substrate while distortions are induced by local fluctuations in the morphology of the substrate (Figure 5B,C).^[^ ^27^ ^]^

Figure 5D,H,L,P and Figure S9, Supporting Information, show that Li seeds nucleated in the electrolyte with low exchange current density are fewer and smaller than those formed with high exchange current density. The top of the Li seed is a hotspot for the further electrodeposition due to the high local current density and thus it is supposed to receive high intensity of electrodeposition (Figures S10 and S11, Supporting Information), leading to faster growth of Li in these locations. With time, a branched structure develops on top of the Li seeds and the evolution of this structure is much faster for high exchange current density, as in the case of the electrolytes [Li(G4)_4_][TFSI] and [Li(G4)_8_][TFSI] (Figure 5L–S). It is worth noting that the development of this structure eventually leads to a dendritic morphology of the Li electrode, with a high porosity that will reduce the Coulombic efficiency of Li as well as potentially lead to internal short circuits.^[^ ^52^ ^]^ In contrast, a dense morphology, with larger granular structures of Li, is obtained at low exchange current density on Li electrode, i.e., in the electrolytes [Li(G4)][TFSI] and [Li(G4)_2_][TFSI] (Figure 5D–K). In particular, the tortuosity of the deposited Li is minimized in the [Li(G4)][TFSI] electrolyte because of the relatively uniform distribution of local current density. The deposited Li with this morphology is ideal to operate a Li metal anode in practice in a rechargeable battery system.

**Figure 6:**
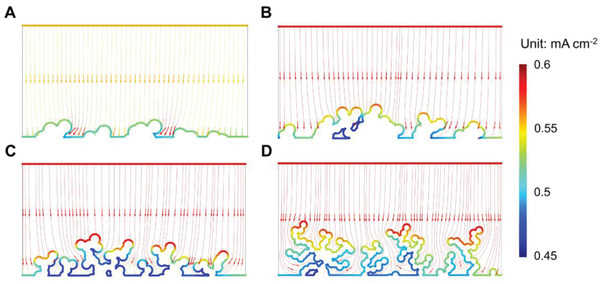
Cross section of electrodeposition of Li in the electrolytes A) [Li(G4)][TFSI], B) [Li(G4)_2_][TFSI], C) [Li(G4)_4_][TFSI], and (D) [Li(G4)_8_][TFSI].

A cross section of the local surface at deposition of Li is acquired to investigate how the physical fields are distorted and promote various electrodeposition behaviors of Li. The electric field near the electrode surface for low exchange current density ([Li(G4)_4_][TFSI]) shows a lower intensity and also is more uniform, providing conditions for even electrodeposition of Li, as seen in **Figure** **6**A. In contrast, the dendritic, branched, morphology distorts the electric field (Figure 6B–D), and the distortion increases with the increase of the exchange current density on Li electrode and further enhances the uneven distribution of local current density. Therefore, a concentration of electrodeposition density on top of dendrites can be expected, leading to a faster growth of a tortuous morphology of Li with increasing number of cavities and pores.

**Figure 7:**
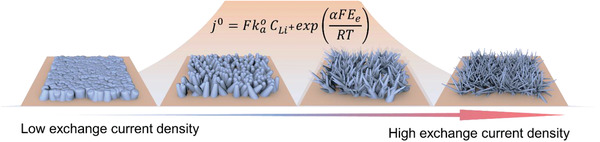
Schematic of electrodeposition behavior of Li under different exchange current densities.

Our results and the discussion above on the reduction, nucleation, and growth mechanisms clearly point to that the electrodeposition behavior of Li is strongly dependent on the exchange current density at the surface of electrode, as shown in **Figure** **7**. Electrodeposition with low exchange current density results in a dense morphology, with low aspect ratio and less columnar Li, while high exchange current density leads to a needle‐like morphology rich of Li dendrites. Therefore, lowering the exchange current density is a clear path to uniform deposition of Li and an improved Coulombic efficiency as well as increased safety. Based on the equation that determines the exchange current density on the electrode

(11) $$j^{0}=Fk_{a}^{o}C_{Li^{+}}\exp\left(\frac{\alpha FE_{e}}{RT}\right)$$

where $k_{a}^{o}$ is the rate coefficient for the reaction Li^+^ + *e* ^−^⇌Li and *E*_e_ is the equilibrium potential of Li^+^/Li against the electrolyte. We can understand the role of the exchange current density in previous reported strategies to enhance the electrochemical performance of Li metal anodes:

1) 3D scaffolds, e.g., metallic foams, fibers, or aerogel, ^[^ ^53^, ^54^, ^55^ ^]^ have been used to prepare composite Li anodes. The design does not actually lead to a lower the exchange current density, but the architecture lowers the real applied current density as a result of the higher surface compared to a 2D structure. Furthermore, it also results in a uniform distribution of local current density on the electrode surface.
2) An artificial SEI, including polymer‐based and inorganic layers with high ionic conductivity,^[^ ^9^, ^56^ ^]^ will accelerate the transport of Li ions near the substrate and thus lower concentration polarization, ($\frac{C_{Li^{+}}^{b}}{C_{Li^{+}}^{s}}$), leading to a lower exchange current density.
3) Li surface modification by alloying with other elements, e.g., magnesium (Mg), aluminum (Al), zinc (Zn), germanium (Ge), or sliver (Ag),^[^ ^57^, ^58^ ^]^ can potentially lower the equilibrium potential and thus enable a decreased exchange current density.

## 3 Conclusions

Phase‐field modeling and experimental characterization are combined to elucidate the critical effect of exchange current density on the electrodeposition behavior of Li in a thermodynamic perspective of Li‐ion reduction and electrocrystallization. We found that the distribution of local current density is more even with a lower exchange current density resulting in a uniform deposition of Li. We show that both the nucleation process and the following growth of Li are directly influenced by the exchange current density. The nucleation of Li with lower exchange current density proceeds with nuclei of larger radius leading to a Li deposition in structures of low aspect ratio. This in turn results in a uniform distribution of local current density and less distortion of the electric field on the surface and an even growth of Li can be expected promoting a morphology with less cavities and pores of the deposited Li. In contrast, a highly branched structure shows up for Li deposited from electrolytes with high exchange current density and the formation of Li dendrites with high aspect ratio invariably leads to the needle‐like morphology with a large porosity. Therefore, we suggest that lowering the exchange current density on the electrode surface is a key to obtain dendrite‐free electrodeposition of Li with a superior interfacial stability and improved Coulombic efficiency. Our work provides a fundamental framework to design novel strategies to accelerate the practical application of Li metal anodes for high‐energy‐density rechargeable battery systems.

## 4 Field‐Phase Simulation Section

The electrochemical reaction for Li deposition can be illustrated as

(12) $$Li^{+}+e^{-}↔\mathrm{Li}\left(s\right)$$

The phase‐field model is based on thermodynamics^[^ ^59^, ^60^, ^61^ ^]^ and the Gibbs free energy of the system can be expressed by

(13) $$G=\int_{V}\left[g\left(C\right)+\frac{1}{2}\left(\nabla \hat{C}\right)\cdot \xi \left(\nabla \hat{C}\right)+\rho_{c}\varphi \right]\;dV$$

where *V* is the volume of the solution unit and *g*(*c*) is the effective bulk free energy density or the Helmholtz free energy density in solution. In our system, *C* = {*C*_1_, *C*_2_, *C*_3_} is the set of concentrations of Li atoms, Li ions and TFSI^−^. *ξ* is the anisotropic gradient coefficient, which is associated with the surface energy. $\hat{C}=\left\{\frac{C_{1}}{C_{S}},\frac{C_{2}}{C_{0}},\frac{C_{3}}{C_{0}}\right\}\;$is the set of dimensionless concentrations, *C*_0_ is the normalized bulk concentration of salt in electrolyte, and *C*_S_ is the site density of Li metal. *ρ*_c_ is the local charge density and *φ* is the local electrostatic potential, so *ρ*_c_ *φ* is the electrostatic energy density. The rate of electrochemical reaction is determined by using the Butler–Volmer expression,^[^ ^59^, ^62^, ^63^ ^]^ from which the local current density can be calculated according to the equation.

In the phase‐field model, the association of exchange current density to the nucleation and growth of Li is built on Li‐ion diffusion equation controlled by multi‐physical field (concentration and electric field) and the emergence algorithm of nucleation based on thermodynamics. In order to reliably simulate the substrate for the electrodeposition process, an electrode surface with a certain roughness is constructed as a function of time. The roughness of the electrode surface change during the deposition process is generated by

(14) $$f\left(x,y\right)=\sum_{m=-M}^{M}\sum_{n=-N}^{N}a\left(m,n\right)\cos\left(2\pi \left(mx+ny\right)+\varphi \left(m,n\right)\right)$$

where *x* and *y* are the spatial coordinates; *m* and *n* are the spatial frequencies; *a* (*m*,*n*) is the amplitude; and *φ* (*m*,*n*) is the phase angle. The amplitude is randomly generated by a Gaussian distribution function, and the phase angle and spatial frequency are derived from a uniform random distribution in a limited interval.

The equation for the conservation of charge in the system is described by

(15) $$\nabla i_{\mathrm{ed}}+\nabla i_{\mathrm{ey}}=0$$

(16) $$\nabla i_{\mathrm{ed}}=-S_{a}j_{n}$$

(17) $$\nabla i_{\mathrm{ey}}=-S_{a}j_{n}$$

where ∇ is Del operator, *i*_ed_ is the electronic current density on the electrode, *i*_ey_ is the ionic current density in the electrolyte, and *S*_a_ is the specific surface area. *j*_n_ is the local reaction current density. The transport of electrons in the electrode follows Ohm's law

(18) $$i_{\mathrm{ed}}=-\sigma_{\mathrm{ed}}\nabla \varphi_{\mathrm{ed}}$$

where *σ*_ed_ is the effective electrical conductivity of the electrode. The transport of Li ions in electrolyte can be expressed as

(19) $$i_{\mathrm{ed}}=-\sigma_{\mathrm{ey}}\nabla \varphi_{\mathrm{ey}}+\frac{2RT\sigma_{\mathrm{ey}}}{F}\left(1+\frac{\partial \ln f_{\mathrm{av}}}{\partial \ln C}\right)\left(1-t_{N}\right)\nabla \left(\ln C\right)$$

where *σ*_ey_ is the effective ionic conductivity of electrolyte, *f*_av_ is the average molar activity coefficient, and *t*_N_ is the transference number of Li ions in electrolyte. *C* is the salt concentration of electrolyte.

The equation for the conservation of mass in the system is described by Fick's law

(20) $$\frac{\partial C_{a}}{\partial t}+\frac{1}{r^{2}}\frac{\partial }{\partial r}\;\left(-r^{2}D_{\mathrm{ed}}\frac{\partial C_{a}}{\partial r}\right)=0$$

where *C*_a_ is the concentration of Li in the electrode material active particles, *t* is the reaction time, *r* is the radial coordinate inside a spherical particle of electrode material, and *D*_ed_ is the diffusion coefficient of Li in the active material.

The electrolyte is described by

(21) $$V_{f}\frac{\partial C}{\partial t}+\nabla \cdot J_{\mathrm{ey}}=\frac{S_{a}}{F}\;$$

(22) $$J_{\mathrm{ey}}=-D_{\mathrm{ey}}\nabla C+\frac{i_{\mathrm{ey}}\cdot t_{N}}{F}$$

where *V*_f_ is the volume fraction of liquid electrolyte inside the porous electrode, *J*_ey_ is the molar flux of Li ions, and *D*_ey_ is the diffusion coefficient of Li ions.

The phase‐field model in our work is simulated by finite element method on the COMSOL Multiphysics 5.5 platform. For the simulation of local current density on the Li electrode, the size of the 3D simulation model is 50 µm × 50 µm. The thickness of electrolyte layer is 40 µm. In order to yield high‐quality results, the model is built by using ultrafine grid division and the maximum grid size is 0.015 µm. Dirichlet boundary conditions are used to solve Equations (14)–(22). The simulation for the growth process of the Li in a 2D model is conducted with model of 10 µm × 5 µm. The three typical interface modes are built by using ultrafine grid division with a maximum grid size of 0.005 µm. The size of the 3D model is 50 µm × 50 µm × 50 µm. Nucleation sites are randomly distributed on the surface. The transfer probabilities of Li ions in all directions are simulated using a random number generator.

## 5 Experimental Section

TEGDME (G4, ≥99%, Sigma‐Aldrich) was dried over molecular sieves (nominal pore diameter 3 Å, Sigma‐Aldrich) and the final water content was 10 ppm, analyzed by Karl Fischer titration. Dried G4 and LiTFSI (99.95%, Sigma‐Aldrich) were mixed in different stoichiometric ratios and stirred at 400 rpm for 24 h. The ionic conductivity was measured from –30 to 50 °C using a Novocontrol broadband dielectric spectrometer. The viscosity and density of electrolytes were determined by a Lovis 2000 M/ME (Anton Paar) viscosity meter.

Li electrodes were cut from Li metal foil (200 µm, Chemetall Foote Corp.). Cu disks were polished and washed with acetone, and subsequently dried for 2 h under vacuum at room temperature. The cycling measurements were performed in symmetrical coin cells consisting of two Li disks, separator (20 µm, Celgard 2400) and electrolytes. Cu disk, separator (20 µm, Celgard 2400) and Li disk were assembled to make a half‐cell for Coulombic efficiency test. The electrochemical measurements were conducted on a multichannel electrochemical analyzer (Ivium‐n‐Stat instrument, Ivium Technologies BV). Cycling measurements of symmetric cells were performed at a current density of 0.50 mA cm^−2^ (1 h charge, 1 h discharge, and 5 min open circuit for each cycle).The half‐cells were discharged galvanostatically (current density, 0.50 mA cm^−2^) for 2 h (Li plating), and were then charged to 1.0 V (Li stripping) at the same current density. Impedance spectroscopy on symmetrical coin cells was performed on a Reference 600 (Gamry Instruments) over the frequency range from 0.01 Hz to 1 MHz with an amplitude of 5 mV.

## Conflict of Interest

The authors declare no conflict of interest.

## Supporting information

Supporting Information
