# Supplementary material for: Insight into the Critical Role of Exchange Current Density on Electrodeposition Behavior of Lithium Metal
Source: Adv Sci (Weinh). 2021 Jan 6;8(5):2003301. doi: 10.1002/advs.202003301 (PMC7927631; doi:10.1002/advs.202003301)
Supplement: Supplementary file 1 — Supporting Information [file ADVS-8-2003301-s001.pdf]

WILEY-VCH

© 2020 Wiley-VCH GmbH

ADVANCED SCIENCE
Open Access

Supporting Information

for *Adv. Sci.*, DOI: 10.1002/advs.202003301

Insight into the Critical Role of Exchange Current Density on Electrodeposition Behavior of Lithium Metal

Yangyang Liu, Xieyu Xu, Matthew Sadd, Olesya O. Kapitanova, Victor A. Krivchenko, Jun Ban, Jialin Wang, Xingxing Jiao, Zhongxiao Song, Jiangxuan Song, Shizhao Xiong*, Aleksandar Matic*.*

Supporting Information

Insight into the critical role of exchange current density on electrodeposition behavior of lithium metal

Yangyang Liu, Xieyu Xu, Matthew Sadd, Olesya O. Kapitanova, Victor A. Krivchenko, Jun Ban, Jialin Wang, Xingxing Jiao, Zhongxiao Song, Jiangxuan Song, Shizhao Xiong*, Aleksandar Matic*.*

Y. Liu, J. Ban, J. Wang, X. Jiao, Prof. Z. Song, Prof. J. Song
State Key Laboratory for Mechanical Behavior of Materials, Xi’an Jiaotong University, Xi'an, 710049, P. R. China.
E-mail address: songjx@xjtu.edu.cn.
X. Xu
Faculty of Materials Science, Lomonosov Moscow State University, Leninskie gory 1, Moscow, 119991 Russia.
M. Sadd, Dr. S. Xiong, Prof. A. Matic
Department of Physics, Chalmers University of Technology, SE 412 96, Göteborg, Sweden.
E-mail address: shizhao.xiong@chalmers.se, matic@chalmers.se.
Dr. O. Kapitanova, Dr. V. Krivchenko
Institute of Arctic Technology, Moscow Institute of Physics and Technology, 9 Institutskiy per., Dolgoprudny, Moscow Region, 141701, Russia.

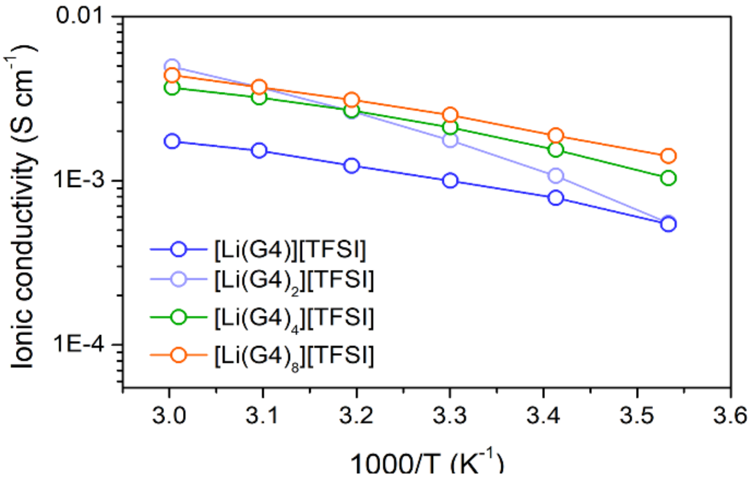

Figure S1. Ionic conductivity as a function of temperature for the different electrolytes.

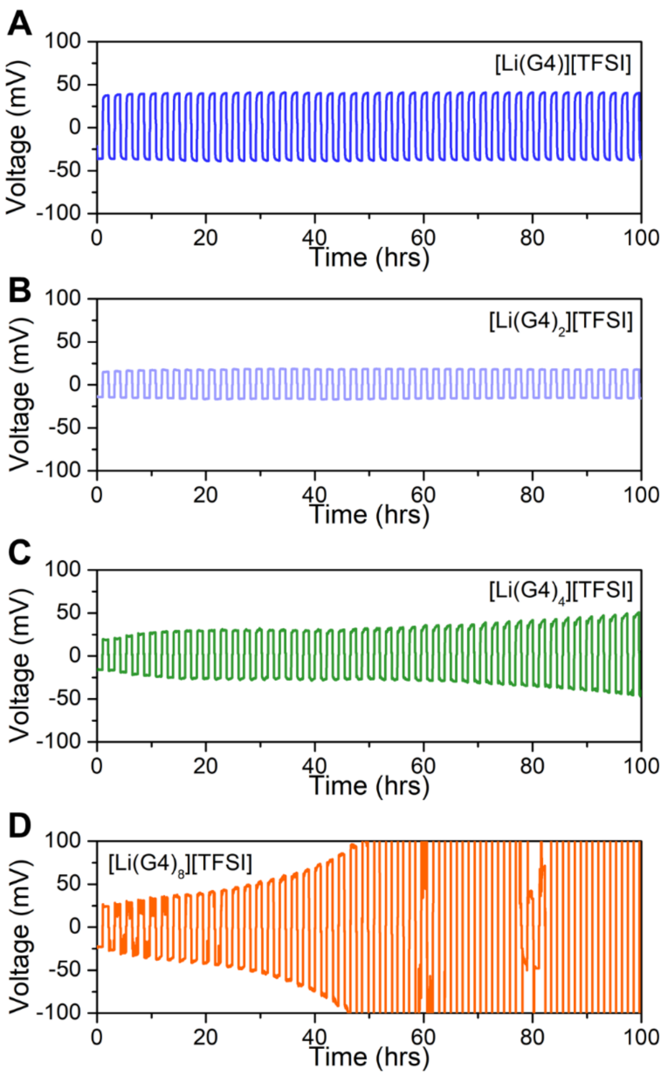

Figure S2. Voltage profiles of Li||Li symmetric cells with electrolytes (A) [Li(G4)][TFSI], (B) [Li(G4)$_2$][TFSI], (C) [Li(G4)$_4$][TFSI] and (D) [Li(G4)$_8$][TFSI].

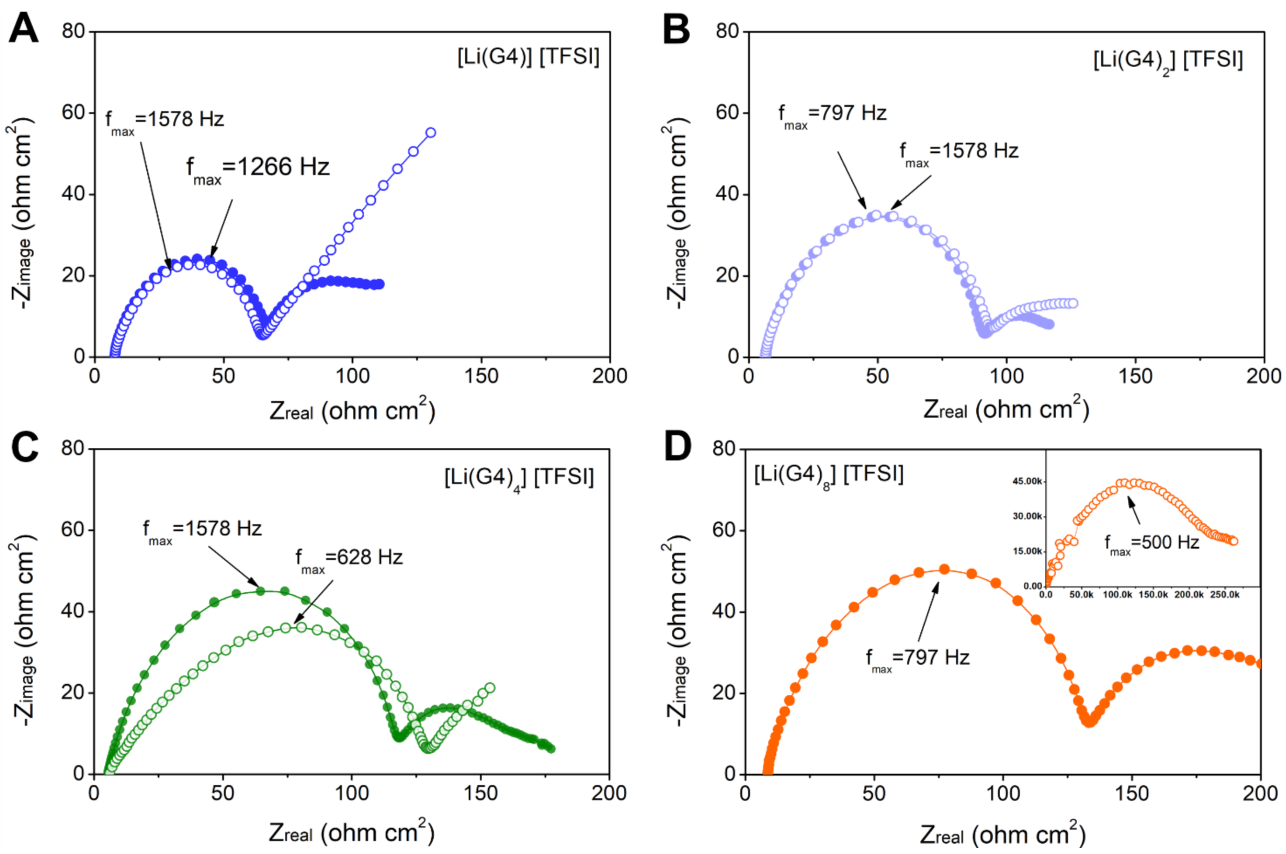

Figure S3. Electrochemical impedance spectroscopy (EIS) of Li||Li symmetric cells before cycling and after 100 hours cycling under a current density of 0.5 mA cm^{-2} with the electrolytes (A) [Li(G4)][TFSI], (B) [Li(G4)$_2$][TFSI], (C) [Li(G4)$_4$][TFSI] and (D) [Li(G4)$_8$][TFSI].

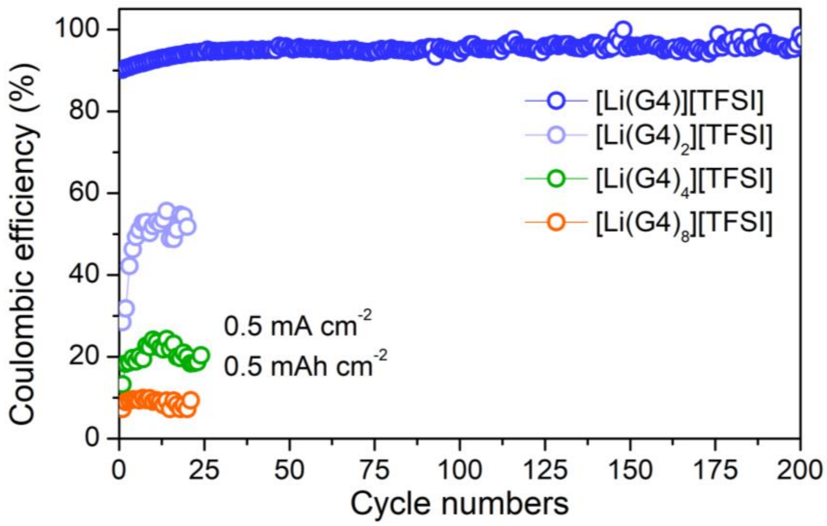

Figure S4. Coulombic efficiency of Li||Cu half cells with different electrolytes at current density of 0.5 mA cm^{-2} and areal capacity of 0.5 mAh cm^{-2}.

Table S1. Physicochemical properties of different electrolytes.

	Mole ratio between G4 and LiTFSI	Kinematic viscosity (mm^2 s^{-1})	Dynamic viscosity (mPa s)	Density (g cm^{-3})	Ionic conductivity (S cm^{-1})	Exchange current density (mA cm^{-2})	Transference number of Li-ion
[Li(G4)][TSFI]	n=1	111.7	157.2	1.4079	0.00107	0.03153	0.55
$[Li(G4)_2][TSFI]$	n=2	34.18	43.4	1.2695	0.00154	0.1001	0.56
$[Li(G4)_4][TSFI]$	n=4	11.43	13.31	1.1646	0.000188	0.5631	0.58
$[Li(G4)_8][TSFI]$	n=8	6.631	7.261	1.095	0.000787	1.2318	0.61

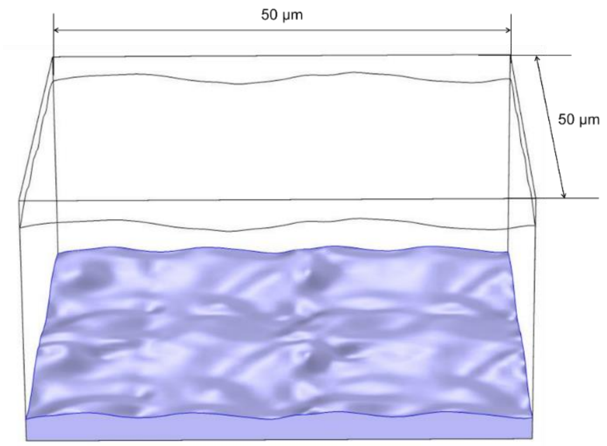

Figure S5. Simulated substrate for Li electrode which is relatively smooth with random morphology.

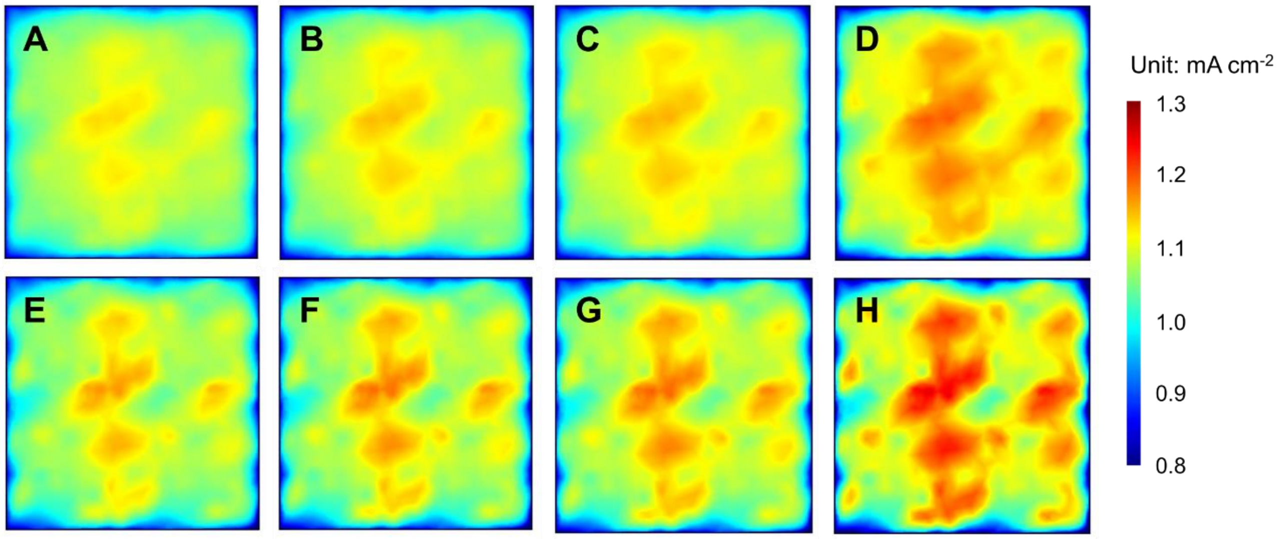

Figure S6. Distribution of local current density on low-roughness Li electrode depositing in the electrolytes (A) [Li(G4)][TFSI], (B) [Li(G4)$_2$][TFSI], (C) [Li(G4)$_4$][TFSI] and (D) [Li(G4)$_8$][TFSI], as well as that on high-roughness Li electrode depositing in the electrolytes (E) [Li(G4)][TFSI], (F) [Li(G4)$_2$][TFSI], (G) [Li(G4)$_4$][TFSI] and (H) [Li(G4)$_8$][TFSI]. The current density was 1.0 mA cm^{-2}.

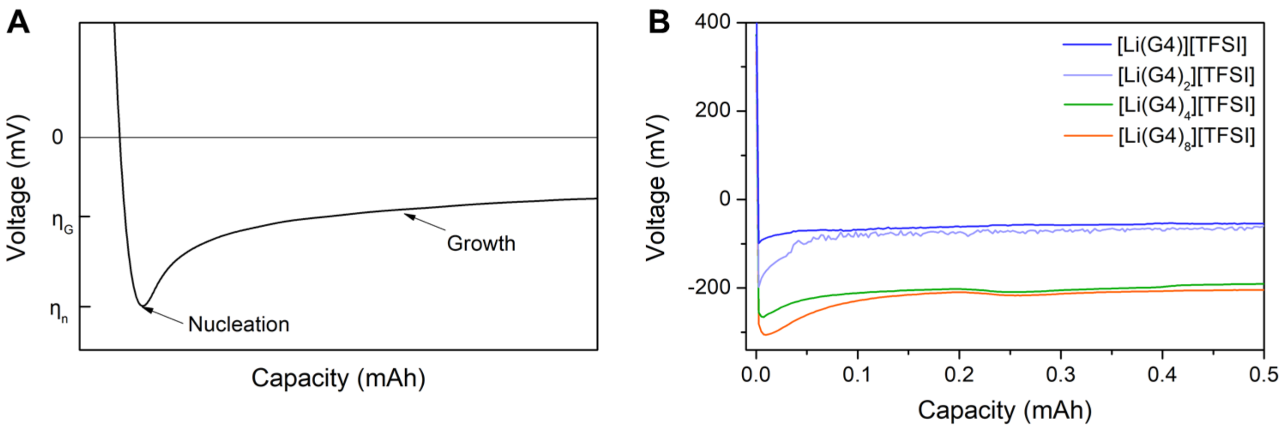

Figure S7. (A) Schematic plot of typical voltage profile of galvanostatic Li plating on Cu. (B) Voltage profiles of Li plating on Cu with different electrolytes.

Table S2. Simulated critical nucleation radius of Li deposited from different electrolytes.

	Substrate 1#	Substrate 2#	Substrate 3#
[Li(G4)][TSFI]	1.77	1.56	1.50
[Li(G4)$_2$][TSFI]	0.95	0.90	0.89
[Li(G4)$_4$][TSFI]	0.78	0.70	0.68
[Li(G4)$_8$][TSFI]	0.67	0.63	0.61

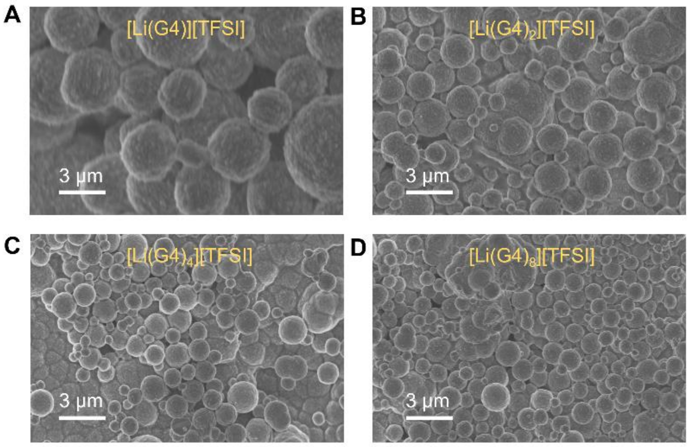

Figure S8 SEM images of deposited Li on copper substrate at 0.5 $mAcm^{-2}$ for 12 min.

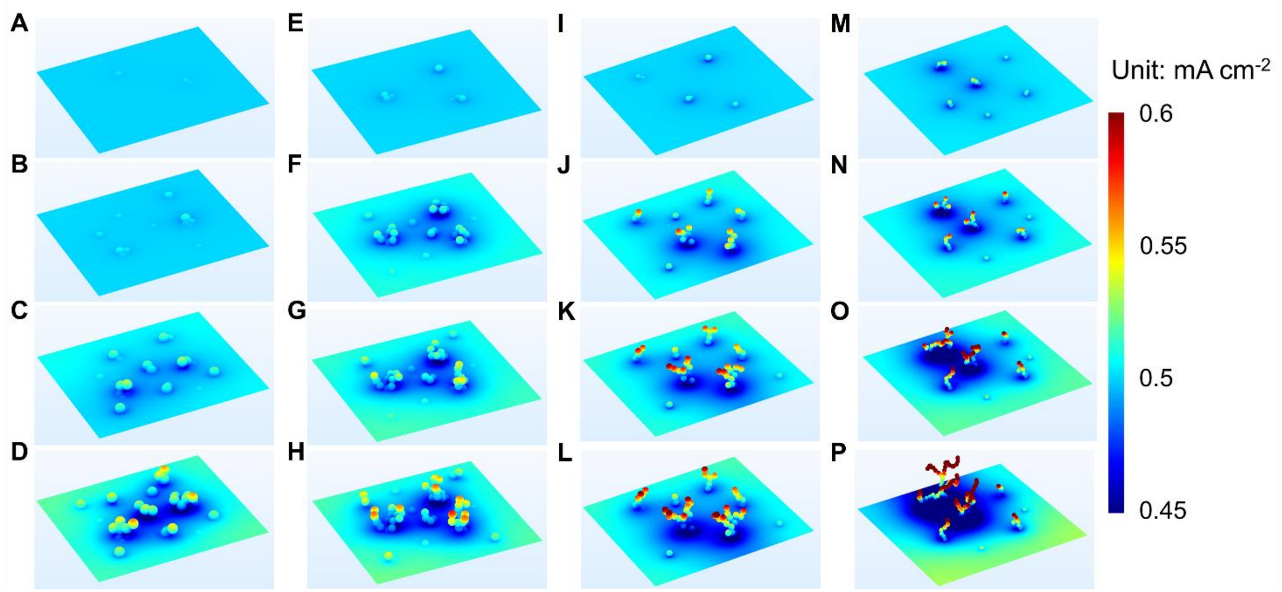

Figure S9. A 45-degree angle view of the simulated morphology with distribution of local current density for Li deposited in the electrolytes (A, B, C and D) [Li(G4)][TFSI], (E, F, G and H) [Li(G4)$_2$][TFSI], (I, J, K and L) [Li(G4)$_4$][TFSI] and (M, N, O and P) [Li(G4)$_8$][TFSI]. The applied current density was 0.5 mA cm^{-2}. Scale bar represents the local current density on the surface of electrodeposition.

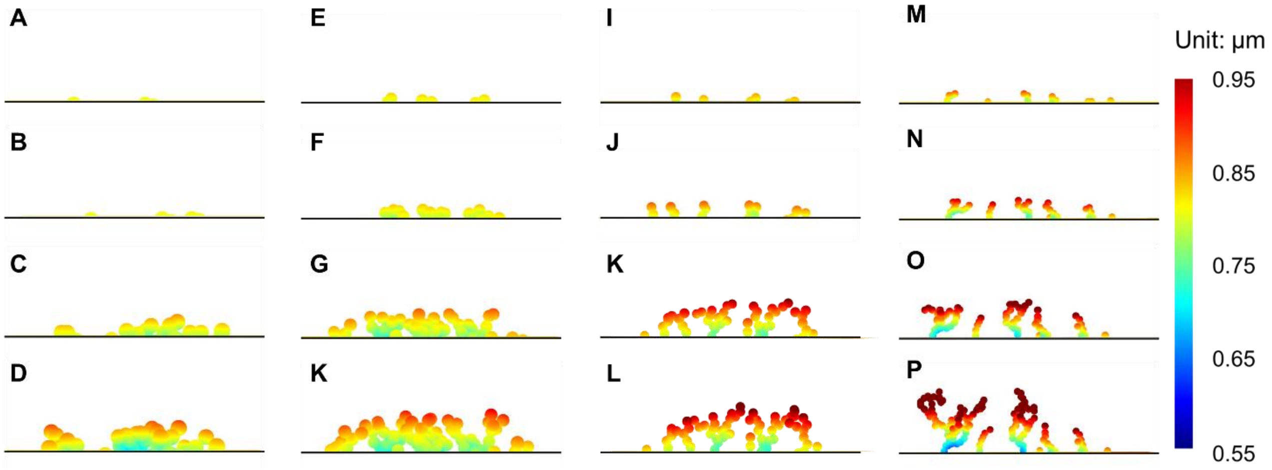

Figure S10. Cross section of simulated morphology with distribution electrodeposition intensity for Li deposited in the electrolytes (A, B, C and D) [Li(G4)][TFSI], (E, F, G and H) [Li(G4)$_2$][TFSI], (I, J, K and L) [Li(G4)$_4$][TFSI] and (M, N, O and P) [Li(G4)$_8$][TFSI]. The applied current density was 0.5 mA cm^{-2}

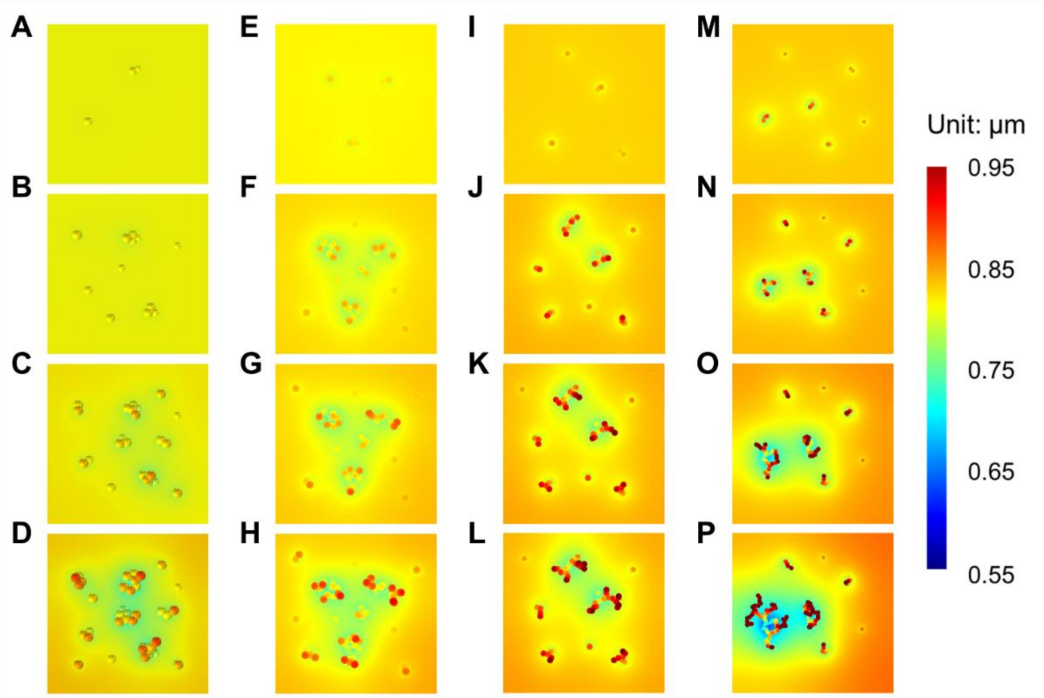

Figure S11. Top-view of simulated morphology with distribution of electrodeposition intensity for Li deposited in the electrolytes (A, B, C and D) [Li(G4)][TFSI], (E, F, G and H) [Li(G4)$_2$][TFSI], (I, J, K and L) [Li(G4)$_4$][TFSI] and (M, N, O and P) [Li(G4)$_8$][TFSI]. The applied current density was 0.5 mA cm^{-2}
